# Supplementary material for: Plant height variation and genetic diversity between Prunus ledebouriana (Schlecht.) YY Yao and Prunus tenella Batsch based on using SSR markers in East Kazakhstan
Source: PeerJ. 2024 Jan 11;12:e16735. doi: 10.7717/peerj.16735 (PMC10788089; doi:10.7717/peerj.16735)
Supplement: Table S1 [file peerj-12-16735-s001.docx]

**Table S1:**

**Geographic locations of collected three populations of *P. ledebouriana* and one population of *P. tenella* in Eastern Kazakhstan.**

| **Population ID** | **Species** | **Region** | **Number of plants** | **20 plants** | | **Location /**  **Altitude (meters above sea level)** | | **Notes** |
| --- | --- | --- | --- | --- | --- | --- | --- | --- |
|  |  |  |  | **Mean plant height** | **Standard deviation** |  |  |  |
| 1-UR | *P. ledebouriana* | Urjar | 20 | 1.78 m | 0.03 | 47°07'17.4"N 81°39'18.5"E | 513m | Near the Urdzhar village |
| 2-KO | *P. ledebouriana* | Kokpekty | 20 | 1.79 m | 0.03 | 49°06' 36.0"N 83°24' 57.6"E | 823m | Near the Samarskoe village |
| 3-KA | *P. ledebouriana* | Katon-Karagay | 20 | 2.08 m | 0.28 | 49°05'58.0"N 84°29'00.8"E | 635m | Near the Kokterek village |
| 4-UK | *P. tenella* | Ulansky | 20 | 1.41 m | 1.04 | 49°57'22.2"N 82°30'06.2"E | 310m | Near the Novo-Akhmirovo village |
